# Our Health Counts: Nothing about us without us in our right to be counted

**DOI:** 10.17269/s41997-024-00928-z

**Published:** 2024-08-08

**Authors:** Raglan Maddox, Sarah Funnell

**Affiliations:** 1 Bagumani (Modewa) Clan, Milne Bay, Papua New Guinea; 2 Yardhura Walani, National Centre for Aboriginal and Torres Strait Islander Wellbeing Research, National Centre for Epidemiology and Population Health, Research School of Population Health, The Australian National University, Canberra, ACT Australia; 3 Unceded Algonquin Territory, Ottawa, ON Canada; 4 Department of Family Medicine, Queen’s University, Kingston, ON Canada

*We call upon the federal government, in consultation with Aboriginal peoples, to establish measurable goals to identify and close the gaps in health outcomes between Aboriginal and non-Aboriginal communities, and to publish annual progress reports and assess long-term trends. Such efforts would focus on indicators such as: infant mortality, maternal health, suicide, mental health, addictions, life expectancy, birth rates, infant and child health issues, chronic diseases, illness and injury incidence, and the availability of appropriate health services.*

Call to Action #19 (Truth & Reconciliation Commission of Canada, 2015).

Change requires our voices, our truths, and our right to sovereignty, self‐determination and agency to transform systems and improve Indigenous health and well-being (United Nations Declaration on the Rights of Indigenous Peoples [UNDRIP]; United Nations General Assembly, 2007). Academic research regarding Indigenous Peoples has been grounded in colonial histories and paternalism noted as early as 1996 by the Royal Commission on Aboriginal People (RCAP; Erasmus & Dussault, 1996). For more than a century, Canada’s Indigenous policies were to eradicate Indigenous governance, ignore Indigenous rights, annul treaties, and promote assimilation with the ultimate aim of erasing Indigenous Peoples across Canada. This was described as “cultural genocide” in the Final Report of the Truth and Reconciliation Commission of Canada (Truth & Reconciliation Commission of Canada, 2015, p.1).

Health researchers have an ongoing history of insufficient collaboration with Indigenous Peoples and communities (Smylie & Firestone, 2015). Standard research approaches have led to mistrust and scepticism by Indigenous Peoples, who, at a population level, have gained limited health improvements as evidenced by ongoing health inequities (Allan & Smylie, 2015). Research has held a poor reputation among Indigenous Peoples, commonly being undertaken “on us” and not “with us” (Smylie et al., 2020). There remains a concern that Indigenous Peoples have been over-researched without corresponding improvements (Funnell et al., 2020), with this trend common among Indigenous populations internationally (Kinchin et al., 2017).

Indigenous Peoples have led and undertaken meaningful and high-quality research since time immemorial, which is evidenced by our continued existence in spite of colonization, and across academic disciplines and fields of expertise. Researchers and institutions are beginning to recognize that Indigenous Peoples and communities are the knowledge holders and have the right to lead research, monitoring, evaluation, and associated program and policy reform. Given existing strengths, rich diversity, and resilience of Indigenous Peoples, Our Health Counts (http://www.welllivinghouse.com/what-we-do/projects/our-health-counts/) embarked on a multi-year learning journey to better understand Indigenous health from the voices of Indigenous Peoples.

Through embracing the principle of self-determination as articulated in UNDRIP (United Nations General Assembly, 2007), Our Health Counts took a “Nothing about us without us” approach to Indigenous health research (Funnell et al., 2020). Drawing on such Indigenous excellence, the overarching goals of the Our Health Counts research program are to:

- enhance the responsiveness, relevance, effectiveness, and efficiency of health and social services for Indigenous populations living in urban and related areas;
- develop sustainable infrastructure that will produce scientifically excellent and community-relevant population-based Indigenous health information;
- develop community-owned and governed comprehensive population health databases that are being actively applied to health and well-being service planning, delivery, and evaluation.

Reflecting ethical requirements and evidence-based wise practices, the Our Health Counts community-partnered research approach “moves beyond, and is distinct from, community-based participation action research in its deliberate application and demonstration of Indigenous paradigms, principles, knowledge, and practices” (Smylie et al., 2024). The Our Health Counts approach upholds Indigenous Data Governance and Indigenous Data Sovereignty Principles, attempting to better balance the power relationships among Indigenous community research partners, academics and additional governmental, data, and public health stakeholders throughout the research process, while maintaining rigour and policy relevance. Clearly negotiated and properly implemented research, data sharing, and publication agreements are critical to Indigenous health research and an area of significant concern and critique by Indigenous stakeholders in Canada. The Our Health Counts methods of Indigenous community research engagement and health data partnership have been tested and refined over time. This includes existing policy-relevant research and data sharing projects with Indigenous health service research partners as outlined in the present CJPH supplement.

Rather than being a resource extraction exercise where academics take from Indigenous communities for their own self-promotion, Our Health Counts privileges community‐led questions moving beyond quantifying risk factors. Our Health Counts upholds the rights of Indigenous communities to be in control of the research. Community control goes beyond involvement and consultation. It emphasizes that Indigenous People have the fundamental right to make decisions regarding research that impacts their community (Funnell et al., 2020). Partnership on this study meant that Indigenous ways of knowing, being, and doing were at the forefront. This research came from within and was developed from the ground up, in partnership with Indigenous communities in urban settings.

The legacy of deficit reporting has restricted our insight into how we can prioritize and actualize policy and practice reform. Walter and Andersen (Walter & Andersen, 2016) outline that the social positioning of non‐Indigenous “owners” of data unintentionally influences the interpretation of data, leading to significant and often foreseeable consequences (Walter & Andersen, 2016). Our Health Counts (re)frames the research questions Indigenous Peoples and communities want. We want to better understand how policy-makers and health services can meet the needs of Indigenous Peoples, not how our Indigenous Peoples can fit into the existing system and services.

Transformational change is required to appropriately meet the needs of Indigenous Peoples in urban settings and across geographies. This change includes accurate and timely population counts, associated program and service resourcing, priority setting, and strategic planning. This is positioned with acknowledgement of the ongoing nature and impacts of colonization and its manifestations. Coloniality continues to affect our families, our community and all our relations through the dispossession of our lands and erosion of power, social structures and community resources, and the embedding of racism within the landscape; this directly and indirectly impacts Indigenous health and well-being. Unique to Canada is the discriminatory and common exclusion of constitutionally recognized First Nations, Inuit and Métis (FNIM) relatives living in urban and related homelands from Indigenous health policy, funding, and programming tables. The purpose of this supplement is to address the failure to recognize rights of urban FNIM. As Indigenous Peoples, we are inherently connected to this research and the associated outcomes. Our Health Counts, the governance, the survey tools and the outcomes reported, embody Indigenous sovereignty, self‐determination, and agency, supporting communities and future generations to thrive.

Indigenous communities have long voiced concerns about our misrepresentation in academic literature, such as that which specifically promotes settler-colonial privilege at the expense of Indigenous knowledges, lived experiences, and realities and, in turn, commonly reinforces racialized logics and associated deficit discourse (Lee et al., 2023). We must move away from colonial practices of research, evaluation, publishing, and sharing of Indigenous stories, knowledges, and perspectives. We can all benefit from fostering safer spaces; honouring and celebrating Indigenous diversity, nations, languages, and knowledges in progressing scientific excellence; and highlighting the significance of our right to be counted, seen, and heard, to ultimately improve health and well-being outcomes for all.

## The foundation of this editorial

This editorial was conceptualized with Indigenous leadership, building on the work of those who have come before us and including but not limited to our own (RM and SF) Indigenous lived experience. The editorial centres on Indigenous interests, rights, and principles, consistent with UNDRIP and ethical standards (Funnell et al., 2020; Schnarch, 2004; United Nations General Assembly, 2007). It emphasizes the importance of acknowledging the relationships, roles, community accountability, and responsibilities that underlie our Indigenous perspectives, recognizing the influence of biases and worldviews (Moreton-Robinson, 2017; Walter & Suina, 2019).

Relationality is a distinct Indigenous social research presupposition and forms the epistemic scaffolding shaping (Moreton-Robinson, 2017) and supporting the possibility for coming to know and generating knowledge(s) in the respective time, place, and land. By privileging and following our logic(s) of knowledge(s), we come to know who we are and who we claim to be, as well as who claims us and how we are connected to our lands. Our being and how relationality informs our Indigenous social research paradigm and worldviews are critical to this editorial and how it was informed. This is a matter of ontology and epistemological consideration, and not merely a matter of identity. Thus, it is foundational to how we approach our research, our evaluation, how we come to know the world, and how we approached this editorial.

As Indigenous Peoples, we have always undertaken meaningful and high-quality research, and it is an honour to write, right, wright, and celebrate Our Health Counts and the ongoing Indigenous excellence that it embodies. In reflecting on the Our Health Counts Research Program, we are reminded that our truths, right to sovereignty, self‐determination, and agency are needed to improve Indigenous health and well-being. This transformation demands courage, wisdom, and time. When we identify ourselves, stating who we are, where we come from, and importantly, our names, we are honouring and acknowledging our ancestors, and the groundwork they established for us to undertake such meaningful work with humility.

It is through a program of research such as Our Health Counts that Indigenous Peoples meaningfully address the call to close the gap in health outcomes between Indigenous and non-Indigenous communities (Truth & Reconciliation Commission of Canada, 2015). 

Marsi, Meegwetch, Nai’wen, Agutoi, Tenkyu tru, Thank you.

Raglan Maddox, Australian National University; CJPH Associate Editor

Sarah Funnell, Queen’s University; CJPH Senior Editor

## Éditorial

*Nous demandons au gouvernement fédéral* *,* *en consultation avec les peuples autochtones* *,* *d'établir des objectifs quantifiables pour cerner et combler les écarts dans les résultats en matière de santé entre les communautés autochtones et les communautés non autochtones* *,* *en plus de publier des rapports d'étape annuels et d'évaluer les tendances à long terme à cet égard. Les efforts ainsi requis doivent s'orienter autour de divers indicateurs* *,* *dont la mortalité infantile, la santé maternelle, le suicide, la santé mentale, la toxicomanie* *,* *l'espérance de vie, les taux de natalité, les problèmes de santé infantile, les maladies chroniques, la fréquence des cas de maladie et de blessure ainsi que la disponibilité de services de santé appropriés.*

Appel à l’action #19 (Commission de vérité et réconciliation) (Truth & Reconciliation Commission of Canada, 2015).

La transformation des systèmes et l’amélioration de la santé et du bien-être autochtones ne peuvent se faire sans nos voix, nos vérités et notre droit à la souveraineté, à l’autodétermination et à l’agentivité (United Nations Declaration on the Rights of Indigenous Peoples [UNDRIP]; United Nations General Assembly, 2007). La recherche universitaire sur les peuples autochtones est ancrée dans les histoires coloniales et le paternalisme, comme le notait déjà la Commission royale sur les peuples autochtones en 1996 (CRPA; Erasmus & Dussault, 1996). Pendant plus d’un siècle, les politiques autochtones du Canada ont éradiqué la gouvernance autochtone, fait fi des droits autochtones, annulé des traités et promu l’assimilation dans le but ultime d’effacer les peuples autochtones partout au Canada. Cela a été qualifié de « génocide culturel » dans le rapport final de la Commission de vérité et réconciliation du Canada (Truth & Reconciliation Commission of Canada, 2015, p.1).

Les chercheurs et chercheuses en santé n’ont jamais suffisamment collaboré avec les personnes et les communautés autochtones (Smylie & Firestone, 2015). Les approches de recherche standard suscitent la méfiance et le scepticisme chez les personnes autochtones, qui, à l’échelle populationnelle, n’ont fait que des gains limités sur le plan de la santé, comme en témoignent les iniquités continues dans ce domaine (Allan & Smylie, 2015). La recherche a mauvaise réputation chez nous, les personnes autochtones, car elle est généralement menée « sur nous » et non pas « avec nous » (Smylie et al., 2020). Il demeure préoccupant que les peuples autochtones soient sur-étudiés sans pour autant que leur situation s’améliore (Funnell et al., 2020), une tendance courante dans les populations indigènes à l’échelle mondiale (Kinchin et al., 2017).

Des personnes autochtones ont de tout temps dirigé et mené des études sérieuses et de haute qualité, comme en atteste leur existence continue malgré la colonisation, et ce, dans l’ensemble des disciplines universitaires et des domaines de compétence. Les chercheurs et chercheuses et les établissements d’enseignement commencent à reconnaître que les personnes et les communautés autochtones sont détentrices de savoirs et ont le droit de diriger la recherche, le suivi, l’évaluation, ainsi que les programmes et réformes stratégiques associés. Étant donné les forces existantes, la riche diversité et la résilience des peuples autochtones, l’équipe du programme de recherche Our Health Counts/Notre santé compte (http://www.welllivinghouse.com/what-we-do/projects/our-health-counts/) a amorcé un parcours d’apprentissage pluriannuel pour mieux comprendre la santé autochtone en écoutant les personnes autochtones.

Adhérant au principe de l’autodétermination tel que défini dans la Déclaration des Nations Unies sur les droits des peuples autochtones (United Nations General Assembly, 2007), l’équipe de Notre santé compte a fait sien le slogan « Rien sur nous sans nous » pour aborder la recherche en santé autochtone (Funnell et al., 2020). Les buts ultimes de Notre santé compte, qui s’appuient sur l’excellence autochtone, sont les suivants :

- améliorer la réactivité, la pertinence, l’efficacité et l’efficience des services sociaux et de santé pour les populations autochtones vivant dans les zones urbaines et connexes;
- créer des infrastructures durables en vue de produire des informations populationnelles sur la santé autochtone à la fois excellentes sur le plan scientifique et pertinentes pour les communautés;
- créer des bases de données de santé exhaustives détenues et gérées par les communautés autochtones et les appliquer activement à la planification, à la prestation et à l’évaluation des services de santé et de bien-être.

Pour tenir compte des exigences éthiques et des pratiques éclairées fondées sur les preuves, l’approche de recherche de Notre santé compte, menée en partenariat avec la communauté, « va au-delà de la recherche-action participative communautaire et s’en distingue par son application et sa démonstration délibérées des paradigmes, des principes, des savoirs et des pratiques autochtones » [traduction libre] (Smylie et al., 2024). L’approche de Notre santé compte défend les principes de la gouvernance et de la souveraineté des données autochtones en essayant de mieux équilibrer les rapports de force entre les partenaires de la recherche communautaire autochtone, les universitaires et les autres parties prenantes du gouvernement, du milieu des données et de la santé publique tout au long du processus de recherche, tout en préservant la rigueur de ces données et leur utilité pour les politiques. Des ententes clairement négociées et dûment appliquées en matière de recherche, de partage des données et de publication sont indispensables à la recherche en santé autochtone et font l’objet de préoccupations et de critiques importantes par les parties prenantes autochtones au Canada. Les méthodes d’implication des peuples autochtones dans la recherche communautaire et de partenariat en matière de données sur la santé employées par Notre santé compte ont été testées et perfectionnées avec le temps, entre autres dans le cadre de projets existants, décrits dans le présent supplément de la RCSP, axés sur la recherche et le partage de données utiles pour les politiques avec des partenaires de la recherche en services de santé autochtones.

Au lieu d’être un exercice d’extraction des ressources de communautés autochtones par des universitaires à des fins d’autopromotion, le programme Notre santé compte privilégie les questions venant de ces communautés, ce qui va au-delà de la mesure des facteurs de risque. Notre santé compte défend le droit des communautés autochtones de contrôler la recherche. Ce contrôle communautaire dépasse la participation et la consultation. Il insiste sur le fait que les personnes autochtones ont le droit fondamental de prendre les décisions de recherche qui ont des répercussions sur leur communauté (Funnell et al., 2020). Dans le cadre de notre recherche, le partenariat signifie la mise au premier plan des façons de connaître, d’être et de faire des peuples autochtones. La recherche est venue de l’intérieur et s’est développée à partir de la base, en partenariat avec des communautés autochtones de zones urbaines.

Le « discours déficitaire» a laissé en héritage une perception limitée des façons dont il serait possible de prioriser et d’actualiser des réformes dans les politiques et les pratiques. Walter et Andersen (Walter & Andersen, 2016) expliquent que la position sociale des « propriétaires » non autochtones de données influence involontairement l’interprétation de ces données, ce qui a des conséquences importantes et souvent prévisibles (Walter & Andersen, 2016). Le programme Notre santé compte (re)formule les questions de recherche réclamées par les personnes et les communautés autochtones. Son objectif est de mieux comprendre comment les responsables des politiques et les services de santé peuvent répondre aux besoins des personnes autochtones, et non pas comment ces personnes pourraient s’ajuster au système et aux services existants.

Un changement transformatif est nécessaire pour répondre convenablement aux besoins des personnes autochtones des zones urbaines et de diverses régions géographiques. Un tel changement inclut des chiffres de population exacts et à jour, des ressources pour les programmes et services associés, l’établissement de priorités et la planification stratégique. Il s’inscrit dans la reconnaissance de la persistance et des impacts de la colonisation et de ses manifestations. La colonialité continue de toucher nos familles, notre communauté et toutes nos relations par l’expropriation de nos terres, l’érosion de nos pouvoirs, de nos structures sociales et de nos ressources communautaires et l’encastrement du racisme dans le paysage; tout cela a des impacts directs et indirects sur la santé et le bien-être autochtones. Le Canada se distingue par son exclusion discriminatoire et ordinaire des personnes apparentées aux Premières nations, aux Inuits et aux Métis (PNIM), reconnues par la Constitution et vivant sur leurs propres terres urbaines et connexes, des tables autochtones sur la programmation, le financement et les politiques de santé. Le présent supplément porte sur l’omission de reconnaître les droits des PNIM vivant en zone urbaine. En tant que personnes autochtones, nous sommes intrinsèquement liés à cette recherche et à ses résultats. Le programme Notre santé compte, la gouvernance, les outils d’enquête et les résultats déclarés incarnent la souveraineté, l’autodétermination et l’agentivité autochtones et aideront les communautés et les générations futures à prospérer.

Les communautés autochtones expriment depuis longtemps leur inquiétude devant leur présentation inexacte dans la littérature savante, celle qui met spécifiquement de l’avant le privilège du colonialisme de peuplement aux dépens des savoirs, des vécus et des réalités autochtones et, en retour, renforce généralement la logique racisée et le discours déficitaire associé (Lee et al., 2023). Nous devons nous éloigner des pratiques coloniales de recherche, d’évaluation, de publication et de partage des histoires, des savoirs et des perspectives autochtones. Nous avons tous intérêt à favoriser des espaces sûrs, à honorer et à célébrer la diversité, les nations, les langues et les savoirs autochtones dans l’avancement de l’excellence scientifique et à insister sur l’importance de notre droit d’être comptés, vus et entendus pour améliorer à terme la santé et le bien-être de tous.

## Le fondement de cet éditorial

Cet éditorial a été conceptualisé sous la direction de personnes autochtones et s’inspire à la fois du vécu autochtone de l’auteur et de l’autrice (RM et SF) et des travaux de ceux et celles qui les ont précédés. Il s’articule autour des intérêts, des droits et des principes autochtones, conformément à la Déclaration des Nations Unies sur les droits des peuples autochtones et aux normes éthiques (Funnell et al., 2020; Schnarch, 2004; United Nations General Assembly, 2007). Il souligne l’importance de signaler les relations, les rôles, les responsabilités envers les communautés et les obligations qui sous-tendent nos perspectives autochtones, tout en reconnaissant l’influence des préjugés et des visions du monde différentes (Moreton-Robinson, 2017; Walter & Suina, 2019).

La relationnalité est une présupposition distincte de la recherche sociale autochtone; elle en forme l’étayage épistémique (Moreton-Robinson, 2017) et soutient la possibilité d’apprendre à connaître et de générer des savoirs qui s’inscrivent dans un temps, un lieu et un territoire donnés. En privilégiant et suivant la logique de ces savoirs, nous en venons à comprendre qui nous sommes et qui nous prétendons être, mais aussi qui fait valoir des prétentions à notre égard et quels sont nos liens avec nos terres. Notre être, et le rôle de la relationnalité dans notre paradigme autochtone en recherche sociale et nos visions du monde, sont essentiels à cet éditorial et aux éléments qui l’ont éclairé. C’est une question d’ontologie et de considération épistémologique et non pas une simple affaire d’identité. La relationnalité est donc à la base de notre approche de recherche et d’évaluation, de notre façon d’en venir à connaître le monde et de notre approche de cet éditorial.

En tant que personnes autochtones, nous avons toujours mené de la recherche importante et de haute qualité, et c’est un honneur pour nous, l’auteur et l’autrice de cet éditorial, que d’écrire, de rectifier, de façonner et de célébrer le programme de recherche Notre santé compte et l’excellence autochtone continue qu’il incarne. Notre réflexion sur Notre santé compte nous rappelle que nos vérités et notre droit à la souveraineté, à l’autodétermination et à l’agentivité sont nécessaires à l’amélioration de la santé et du bien-être autochtones. Une telle transformation exige du courage, de la sagesse et du temps. Quand nous nous identifions, que nous déclarons qui nous sommes et d’où nous venons, sans oublier nos noms, nous honorons et nous reconnaissons nos ancêtres et les jalons qu’ils et elles ont posés pour nous permettre d’entreprendre un travail aussi important avec humilité.

C’est par l’entremise d’un programme de recherche comme Notre santé compte que les personnes autochtones répondront valablement à l’appel à combler l’écart entre les résultats cliniques des communautés autochtones et non autochtones (Truth & Reconciliation Commission of Canada, 2015).

Marsi, Meegwetch, Nai’wen, Agutoi, Tenkyu tru, Thank you.

Raglan Maddox, Australian National University; Rédacteur associé, RCSP

Sarah Funnell, université Queen’s; Rédactrice scientifique adjointe, RCSP
